# Neurorights in the Constitution: from neurotechnology to ethics and politics

**DOI:** 10.1098/rstb.2023.0098

**Published:** 2024-10-21

**Authors:** Sergio Ruiz, Luca Valera, Paulina Ramos, Ranganatha Sitaram

**Affiliations:** ^1^ Psychiatry Department, Interdisciplinary Center for Neurosciences, Medicine School, Pontificia Universidad Católica de Chile, Diagonal Paraguay 362, Santiago de Chile 8330074, Chile; ^2^ Laboratory for Brain-Machine Interfaces and Neuromodulation, Pontificia Universidad Católica de Chile, Diagonal Paraguay 362, Santiago de Chile 8330074, Chile; ^3^ Department of Philosophy, Universidad de Valladolid, Plaza Campus Universitario, Valladolid 47011, Spain; ^4^ Bioethics Centre, Pontificia Universidad Católica de Chile, Diagonal Paraguay 362, Santiago de Chile 8330074, Chile; ^5^ Multimodal Functional Brain Imaging and Neurorehabilitation Hub, Diagnostic Imaging Department, St. Jude Children’s Research Hospital, 262, Danny Thomas Place Memphis, TN 38105, USA

**Keywords:** neurorights, neurotechnology, neuroethics, epistemology, Constitution, Chile

## Abstract

Neuroimaging technologies such as brain–computer interfaces and neurofeedback have evolved rapidly as new tools for cognitive neuroscience and as potential clinical interventions. However, along with these developments, concern has grown based on the fear of the potential misuse of neurotechnology. In October 2021, Chile became the first country to include neurorights in its Constitution. The present article is divided into two parts. In the first section, we describe the path followed by neurorights that led to its inclusion in the Chilean Constitution, and the neurotechnologies usually involved in neurorights discussions. In the second part, we discuss two potential problems of neurorights. We begin by pointing out some epistemological concerns regarding neurorights, mainly referring to the ambiguity of the concepts used in neurolegislations, the difficult relationship between neuroscience and politics and the weak reasons for urgency in legislating. We then describe the dangers of overprotective laws in medical research, based on the detrimental effect of recent legislation in Chile and the potential risk posed by neurorights to the benefits of neuroscience development. This article aims to engage with the scientific community interested in neurotechnology and neurorights in an interdisciplinary reflection of the potential consequences of neurorights.

This article is part of the theme issue ‘Neurofeedback: new territories and neurocognitive mechanisms of endogenous neuromodulation’.

## 1. Introduction

In recent years, in tandem with the growing capacity of neurotechnology to access neural information, concern has grown among certain scientists and political groups that neural data could be used for purposes other than medical or scientific advances. This current of thought warns us that, without regulation, further developments in decoding brain activity could open a Pandora’s box, and that ‘we must act to protect the human mind, before it is too late’ [1]. This updated version of the fear of technology reflects deeply rooted concerns about losing our fundamental attributes as human beings such as our liberty, privacy and agency, and seems to be only matched by current concerns about the vertiginous development of artificial intelligence [2].

Following the rapid advances of neurotechnology for neural decoding and stimulation in the form of brain–computer interfaces (BCIs) and neurofeedback (NF), the term neuroethics was born just a few decades ago [3,4]. It is defined as ‘the examination of what is right and wrong, good and bad about the treatment of, perfection of, or unwelcome invasion of and worrisome manipulation of the human brain’ [5, p. 2]. The term neurorights was coined at the beginning of this century, with pioneering works by Boire and Sententia [6,7] together with the right to ‘cognitive freedom,’ and its first use by Ienca & Andorno [8]. These authors stated that ‘new neuroimaging technologies pose a fundamental ethical and legal and social challenge: determining whether, or under what conditions, it is legitimate to gain access to or to interfere with another person’s neural activity’ [9]. In 2017, Dr Rafael Yuste and the Morningside Group published a highly influential paper on the ‘risks of neurotechnologies and artificial intelligence’ that began with a hypothetical example: a BCI system injured a user, and we were invited to reflect on the problem of ‘agency’ in this hypothetical crime. The article expressed concern about the insufficiency of current ethical guidelines to address these emerging phenomena and advocated for including neurorights in international legal systems and treaties [10]. These new rights usually include the right to: personal identity; free will and mental privacy; equal access to mental augmentation and protection from algorithmic bias. Neurorights raise questions from the perspective of several disciplines including philosophy, ethics, medicine, neuroscience, law and politics. A crucial question, as posed by Ienca, is *‘*to determine whether neurorights should be interpreted as rights in the philosophical sense (moral rights), as rights in the sense of international human rights law (legal rights), or all the above’ [11]. By enshrining neurorights in the Chilean Constitution in 2021, Chilean legislators seem to have been the first to decide how to respond to this extremely complex question.

Yet, the implementation of the constitutional norm has not been exempt from criticism [12,13]. This article briefly explains the current use of those neurotechnologies and the process by which neurorights came to be part of the Chilean Constitution. Although neurorights should encompass all current and new neuromodulation technologies, we will focus on the most targeted neurotechnologies in this debate, namely, BCIs and NF. The second part discusses some of the problems raised by neurorights, in the hope that this discussion will be useful to the scientific community interested in neurotechnologies. The first problem posed is ‘philosophical’, dealing with several epistemological issues raised by the concept of neurorights. The second potential problem is ‘practical’ in nature and is based on the local Chilean experience of implementing similar laws and the threat posed to medical research and practice by over-protective legislation. While these problems belong to different areas, we believe that they represent important areas which illustrate the complexity of this discussion and help separate the wealth of confusing aspects of this phenomenon. Moreover, the philosophical concern is closely related to the practical problem since an epistemological problem in conceptualization can generate negative consequences for legislation—in this regard, Chilean legislation in neurorights may be an interesting example of this powerful connection.

### (a) Brain–computer interfaces and neurofeedback

Our ability to study the human brain has advanced extraordinarily since the first electroencephalographic (EEG) recording in a human almost exactly 100 years ago [14]. Since then, different milestones have marked a century of astonishing cumulative technical progress that has made it possible to greatly improve our understanding of the neural processes that underlie human behaviour. Neurorights may include (or usually do not distinguish) both neurotechnologies that ‘decode’ or ‘monitor’ neural activity, and those that ‘stimulate’ neural activity, also known as neuromodulation [15]. As examples of both aspects of neurotechnologies, we will describe two methodologies: brain–machine interfaces (BMIs)/BCIs and NF, probably the neurotechnologies most frequently mentioned and potentially relevant in discussions of neurorights. A BMI can be described as ‘a reciprocal link between an animal or human brain and an artificial actuator, such as a virtual or robotic arm or leg, for the purpose of utilizing its own brain activity to volitionally control the movements of the actuator, while receiving continuous feedback information from it’ [16, p. 770]. At the turn of the century, BCIs were popularized by researchers at the University of Tubingen in Germany, to decode brain information for communication in locked-in patients [17]. BCIs and BMIs are similar terms, although BCI tends to be used to refer to a BMI in humans, instead of in other primates [18]. A BCI can be defined as a ‘communication system in which messages or commands that an individual sends to the external world do not pass through the brain’s normal output pathways of peripheral nerves and muscle’ [19, p. 769]. Both invasive and non-invasive BCIs exist, which record electrical signals from brain-implanted electrodes or measure electrical and/or haemodynamic activity from outside the brain boundaries. BCIs have used motor intention-related information encoded in the motor cortex to help patients achieve gross motor skills, such as reaching, grasping or moving a computer cursor, for using computer-assisted spellers, wheelchairs and exoskeletons that restore walking [20]. Probably the best-known use of BCIs apart from motor rehabilitation is its use on patients with amyothrophic lateral sclerosis (ALS), a progressive disease that destroys the motor system, although it affects sensory and cognitive functions to a lesser degree. In advanced stages, patients who are perfectly aware of their surroundings find themselves unable to communicate in any way with their external environment, owing to the loss of control of their musculature (i.e., in a ‘locked-in state’ [21]). In a groundbreaking study after decades of development, it was recently demonstrated that speech articulation and other signals can be decoded from intracranial recordings to restore communication even in completely locked-in patients [22].

BCI and NF are related concepts. In both, brain activity must be acquired. However, unlike a BCI, in which brain activity is used to activate an external device, NF uses brain activity essentially as feedback for the user, allowing the learning of self-regulation of the putative neural substrates that underlie a specific behaviour or brain abnormality [23]. Therefore, NF can be conceptualized as a form of *endogenous* brain stimulation and it sits alongside other (exogenous) stimulation neurotechnologies (both non-invasive and invasive) such as transcranial direct current stimulation, transcranial magnetic stimulation, spinal cord stimulation, implanted brain stimulation, electroconvulsive therapy, optogenetics, among many others. Depending on the neural information used, several types of NF exist (electroencephalography (EEG)-NF, functional magnetic resonance imaging (fMRI)-NF, functional near-infrared spectroscopy (fNIRS)-NF, magnetoencephalography (MEG)-NF, etc.). An NF modality used in a rapidly growing research area in recent times is based on real-time fMRI (rtfMRI-NF) [24,25]. This methodology profits from the high spatial resolution and whole brain coverage of fMRI, by measuring changes in the blood oxygenation level-dependent signal [24]. To give only a few examples, rtfMRI-NF has allowed the modulation of several brain areas, including amygdala [26], insula cortex [27], frontal cortex [28], motor areas [29], deep brain structures such as the dopaminergic midbrain [30], among others. As these brain regions are involved in a variety of cognitive processes, NF has been explored increasingly as a potential therapeutic approach in several brain disorders [31,32], including psychiatric disorders (depression [33,34], addiction [35], post-traumatic stress disorder [36,37]) and neurological diseases (e.g. Parkinson’s disease [38,39]), among others. New advances in NF include the use of multivoxel pattern analysis for the decoding and feedback of ‘brain states’ to the subject [40], and the use of decoded neurofeedback (DecNef) [41].

It is important to note that alongside the scientific and clinical use of these neurotechnologies, a growing private industry-led market has appeared. Commercial NF uses include neural information from a subject that promises to track workers’ fatigue levels and mood swings, among other cognitive processes. However, the effectiveness of these devices for clinical purposes remains controversial, as they may lack the methodological rigour and the precision required to decode, and feedback, meaningful neural data [42].

Similarly, while in general the chances of misusing neurotechnology are ever-increasing when combined with information and communication technology and artificial intelligence, NF can present several obstacles to this possibility. As mentioned, NF presents promise for clinical treatment. However, the field still faces several challenges. Studies have shown great variability among participants in learning brain self-regulation with NF, and many participants fail to learn self-regulation [23]. Hypothesized changes in behaviour or symptom improvement do not necessarily correlate with NF training-induced changes in brain activity [43]. Furthermore, the observed behavioural changes or disease improvements typically do not persist over time. The NF research community often attributes the above problems to the prevalent short training regimens and calls for more controlled studies and clinical trials, more specific instructions to the participants for carrying out mental imagery, better feedback and reward contingencies, more sophisticated multimodal and computational tools and techniques [44–46]. Researchers recognize that the neural mechanisms underlying neurofeedback learning are only beginning to be understood. Most importantly, neurofeedback requires the voluntary and continued engagement of participants to understand the tasks and perform them consistently to bring about changes in their brains for neuroplastic and behavioural changes to ensue. Long training regimes are a necessary part of training, just like other forms of clinical rehabilitation.

Although groundbreaking studies have shown the possibility of neural modulations by NF without awareness of the training by the participant [47], these complex NF paradigms still require the engagement of the participant in the task, the presentation of contingent reinforcement and reward proportional to their brain activity. Therefore, although the scenario of a malicious implementation of NF or BCI in an unsuspected participant is not impossible, the successful implementation of such a complex technology (particularly out of the research environment), and its use to produce significant behavioural modifications are quite difficult.

### (b) The path of neurorights in the Chilean Constitution

The process that culminated in the inclusion of neurorights in the Chilean Constitution began in 2019. Neurobiologist Dr Rafael Yuste was invited to participate in person that year in the panel ‘Humano y maquina, una dupla poderosa’ (‘Humans and machine, a powerful duo’) at the Congreso Futuro (Future Congress), an annual event open to the general public to discuss future technologies, coordinated by the Chilean Senate’s Committee on Future Challenges, Science, Technology and Innovation. During his trip, Dr Yuste interacted with politicians in charge of the event and ‘alerted them to the risks associated with neurotechnologies, such as that it was now possible to read emotions, thoughts and the unconscious directly in the brain’ [48]. During the months that followed, Dr Yuste worked jointly with Chilean politicians and Chilean university scholars (including the authors of this article) to discuss the need to establish new fundamental rights, or neurorights. In these meetings, disagreements were expressed over the sense and the scope of neuroprotection as a new human right [49], including the potential ‘redundancy’ of the concept of protecting neurodata, given that privacy as a human right was already protected by our Constitution [50]. Some of these divergent views were expounded by various Chilean and foreign researchers [51,52].

Despite these reservations, the legislative advance of neurorights continued, driven by legislators associated with the aforementioned committee. It is possible that the idea of legislating on neurorights in Chile was fuelled also in part by political contingencies, the particular *Zeitgeist* at the time. On 18 October 2019 (‘*el Dieciocho de Octubre*’, also known as *18-O*), part of the population in our capital Santiago rebelled against the government of President Sebastián Piñera. What came to be known as the *estallido social* (social outburst) was a protest movement that generated episodes of violence, looting and arson, although one week later, on 25 October 2019, over 1.2 million people took to the streets of Santiago to protest peacefully against social inequality. As a result, on 15 November the Chilean National Congress signed an agreement to hold a national referendum on rewriting the Constitution, so that it would be representative of a democracy based on social and economic as well as political rights, a process that is still inconclusive (and that naturally has its supporters and detractors). For some politicians and legislators, this was the opportunity for a ‘maximalist constitution’ [53] that would frame all possible rights and leave no stone unturned. It is possible that this spirit and contingency acted as a catalyst for some politicians and legislators and contributed to the attractiveness of neurorights in Chile.

Finally, in October 2021, neurorights were introduced in Chile by the approval of an amendment to the Chilean Constitution. It consisted of a reform of Article 19 [1] to protect mental integrity and immunity against the adverse effects of neurotechnologies [54], and a process began to draft legislation to regulate neurorights and the development of research and neurotechnologies [55]. The first draft, the comments of the scientists, politicians and lawyers invited to discuss it, and the final law approved by the Chilean Senate, can be found in Annex 1 (see the electronic supplementary material). According to the new constitutional text, ‘Scientific and technological development shall be at the service of people and shall be carried out with respect for life and physical integrity. The law shall regulate the requirements, conditions, and restrictions for its use on persons and with special safeguards for cerebral activity as well as the information deriving from it*’* [55]. Some of its points are summarized below. In its first article, it aims at preserving human physical and psychic integrity in the development and application of neurosciences and neurotechnologies, while recognizing the freedom to use these technologies within the limits established in the Constitution and the law. The bill recognizes three main uses of neurotechnologies: medical uses, the development of research and commercial purposes, giving each one a different regulatory treatment. Any neurotechnology proposed for use in Chile must be registered with the Institute of Public Health, an independent medical authority similar to the United States Food and Drug Administration that is responsible for registering all medicines and devices used for medical purposes. In the registering process, all intended uses of the technology must be indicated and the authority may restrict or even prohibit these uses when: (i) their objective is to influence human conduct without the user’s consent; (ii) they are used to exploit the weaknesses of specific groups; (iii) to extract data without the user’s explicit consent; or (iv) they adversely affect the neuroplasticity of certain groups, especially of children and young adults. On the question of consent, together with the freedom to use any approved neurotechnology, the legislative proposal establishes a differentiated system that considers the intended use. Thus, therapeutic and scientific research uses must follow the regular rules applicable to consent on these matters (*Law 20.584 on the rights and duties of patients*, which will be discussed in §2b of this article), while other, commercial uses, require specific consent granted prior to the technology’s implementation. Consent must be freely given on the basis of information, must be in a written format and be specific to the application in question. As for the eventual damage caused by the use of neurotechnologies, the bill retains the general system of responsibility in scientific research and therapy, but it also establishes a special regime for responsibility when these technologies are used in a commercial context for end-users. In commercial uses, the producer, importer and administrator of the neurotechnologies that caused the damage are jointly and strictly responsible for any damage caused to a consumer. This implies that the victim can sue any of them for the adverse consequences suffered, considering their geographical proximity and economic capacity.

## 2. Potential problems of neurorights

### (a) The epistemological basis of neurorights

Although novel and attractive to the public, the Chilean amendment concerning neurorights must be clarified from the epistemological point of view. Authors have recently discussed the ontological substratum of the Morningside Group’s proposal [10] and offered a more suitable normative, ethical and legislative reasoning on this point [11]. To the best of our knowledge, very little has been argued from an epistemological point of view, even though such an analysis should be a pre-requirement for any legislation in this respect: without a precise examination of some preliminary issues (such as the difference between neural and mental data; the definition of the concepts used in the major amendments and the distinction between moral values and rights), it seems impossible to adequately address neurorights.

In this regard, it is worth distinguishing in advance between neuromodulation and neuroimaging, as two different fields of application of neurotechnologies, with different consequences and problems [56]. Indeed, different ethical concerns emerge when talking alternatively of brain reading techniques [57] and brain modulation interventions, or of devices that can monitor brain activity versus those that can intervene in it [58]. Whereas neuroimaging provides a ‘snapshot’ of neural data—i.e., they play a ‘passive’ role concerning the person involved—neuromodulation actively intervenes in neural circuits, directly modifying the person’s own behaviour, affecting his/her agency.

Depending, thus, on the impact of those neurotechnologies on the human brain, different kinds of ethical assessments would be required of the *habeas mentem* [59,60], and different legal consequences would flow from it. Indeed, ‘neurotechnologies provide the capability to: (i) access someone’s mental states; (ii) verify subjective (or first-person) reports regarding the nature and content of those states; (iii) contest first-person authority regarding mental states by overriding such introspective reports; and (iv) control decoded mental states by providing input behaviourally or through direct brain stimulation’ [58]. Owing to the different impacts on the human brain, each of these activities involves a different and specific ethical assessment.

#### (i) The supposed urgency of neurorights: three main reasons

Why, then, is the legislation regulating current neurotechnological developments needed? What are the causes of such urgency? Or is this legal action premature? Has Chile legislated to protect rights that are under threat only in science fiction [61]? Such questions are not senseless: indeed, various authors [12,53] argue that ‘the current Chilean neurorights reforms are vague and premature’ and ‘consequently, lobbying on their behalf should cease.’ [12, p. 2].

Reconsidering recent debates in the neuroethics area, Bublitz [12] points out three main drivers for such urgency: (i) neuroessentialism, (ii) neuroexceptionalism, and (iii) neurosciencefictionism or neurohype. Neuroessentialism is the tendency to find ultimate explanations for behaviours at the level of neural processes [62–64]. It is a form of reductionism about mental states, experiences and, more generally, the self [65]: if there is a strong relationship between the brain and the different manifestations of personality, we cannot say there is a coincidence or superimposition. This tendency is quite present in papers supporting neurorights or in the proposed amendments themselves: since our humanity is the product of our brain activity, it is argued that any technology that alters our cognitive capabilities will affect our humanity. Consequently, protecting our brain activity ultimately means defending humanity. Such a form of reductionism is built on a logical fallacy (the ‘mereological fallacy’ [66], i.e., ‘the fallacy of ascribing psychological attributes to parts of an animal that can only intelligibly be ascribed to the animal as a whole’ [67, p. 1079]). This kind of fallacy—i.e., a logical mistake—attributes a property to a specific part of a whole, when that property refers to the emergence of the totality of parts (i.e., the whole). In our particular case, it is a mereological fallacy to argue that the brain thinks, feels or listens, whereas it is the whole person who carries out all these activities [67].

The second reason for neurorights—i.e., neuroexceptionalism [68]—mainly deals with a political trend that considers any data or expression of the brain as something exceptional, thus deserving a unique ethical and legal consideration [69]. This is owing to two different forms of claim: ‘i. The claim that neurodata is exceptional. ii. The claim that the exceptional character of neurodata mandates specific and novel legal rules as a response’ [70]. We should then ask ourselves in which respect neurodata is something exceptional compared with other sources of data that we release on a daily basis [12]. At the basis of neuroexceptionalism there is the (meta)physical assumption that we are our brain, while our person clearly exceeds one (or more) organs—this statement is particularly relevant for different bioethical matters, like brain death and organ donation [71].

The third reason for neurorights—i.e., ‘neurosciencefictionism’ or ‘neurohype’ [72]—is crucial to understand the current legislations on neurotechnologies. Although theoretically, it is the weakest reason—indeed, it mainly appeals to emotions and fear of the unknown—it is also the most convincing at the level of the ‘non-specialized’ public. The way it works is this: neuroscientists may magnify the eventual consequences or possibilities of a given scientific discovery to attract the attention of citizens and legislators. In this sense, it is common to read news reports about the almost *Black Mirror*-like possibility of completely transcribing a patient’s thoughts via neurotechnologies, knowing everything he/she wants or his/her most intimate memories. The next step is the need to protect that realm of inscrutable intimacy, which would be exposed to the public if such neurotechnologies were imprudently used. However, the source of the real danger, as Wexler [73] points out, is elsewhere: ‘The data that we generate on a daily basis (for example, e-mail messages, browser queries, passwords, financial information, call data and GPS information) are far more revealing than EEG is now and will be in the foreseeable future’ [73, p. 990]. We are not assuming that all neuroscientists are carelessly sensationalizing to have an impact on the political field, nor does it mean that neuroscience (or neuroethics) is inaccurate. In this sense, Wexler’s article is particularly relevant as it scientifically criticizes the neurohype approach [74]. This shows that we should develop ethical and political reflections starting from real and not fictitious data or unsupported predictions (the ‘neurohype narrative’ [56]). In this sense, we disagree with van de Werff *et al*. [75], when they argue that ‘reducing neurohype is not feasible, and also not always desirable. Neurohype allows scholars in the humanities to ethically reflect in an early stage of scientific development when such reflections could possibly still fuel back into the scientific process. “Reducing” neurohype would then obscure a fruitful venue for productively questioning the ways knowledge of the brain is and should be made valuable in science and society’ [75, p. 98]. Contrary to this, we believe that ‘anticipatory ethics’ should ‘separate speculation from science’ [73]. In this sense, the different propositions to legally protect the human mind from possible intrusion must be based on epistemological humility, that is, the ability to adhere strictly to scientific reality. Moreover, an essential element of such humility is recognizing the different levels and hierarchies of knowledge and appropriately conveying them to the non-expert audience, without improperly mixing them up.

#### (ii) Are we (really) sure about what (some) scientists are telling us?

The previous reflections should have shown that neurorights lies at the crossroads of science and politics, and what is at stake is the influence the former should have on the latter and vice versa. In this regard, ‘while legal regulation should, of course, be anticipatory, it should also be based on a realistic assessment of powers and potentials of technologies’ [12]. It is worth noticing that we are legislating about ‘possible states of affairs’, and thus the question of predictability of technological developments comes into play. The real epistemological question concerns the credibility of scientific predictions and, more precisely, the timescale of those predictions—it is quite different to legislate about the effects and use of neurotechnologies in the next five, or the next fifty years. Indeed, Ratto Trabucco argues: ‘Legal and ethical considerations should be based on proven scientific evidence and realistic predictions, avoiding anti-technology narrative strategies driven by the fear that a transparent discussion on the issue might delay scientific innovation and cancel BCI-associated benefits for people who really need them’ [57, p. 756]. It is worth stressing the importance of realistic predictions—which become more complex with the implementation of AI—that should reasonably consider how the rapidly developing methods may change the landscape.

Can fear, then, drive politics? Is it appropriate to be guided only by the Jonasian heuristics of fear [76,77], in order to stop the ‘unbound Prometheus’ and his ‘immodesty’ [76] caused by the novel technological power? As Marvin brilliantly points out, technological developments have always gone hand-in-hand with apocalyptic prophesies, catastrophes, concerns and public resistance [78]. To avoid unfounded fears—following the precautionary principle—it is appropriate to constantly re-examine data, starting from up-to-date scientific discoveries. This is something that the scientists themselves must carry out in their complex relationship with the non-specialized public and those who make public policy decisions [79].

#### (iii) Concepts and language: epistemological concerns

A relevant part of that dialogue between scientists and the political world is the use of language and concepts. There is a gap between the world of science and politics, between researchers and citizens, that should not be filled completely. These two separate levels should never be mixed: politics must sometimes interact with science, but not always; on the other hand, scientists must strive to be understood by the world of politics (and vice versa!) with appropriate concepts and language, but without the need to abandon their field of expertise. This is part of the epistemological humility scientists should have. An interesting article written by one of the protagonists of the neurorights proposal, ‘Neurotechnologies cannot seize thoughts: a call for caution in nomenclature’, argues: ‘As scholars, we should choose language that accurately reflects the scientific facts, precisely describes the ethical challenges unique to a given context and technique, and depicts a realistic vision of the future’ [80, p. 25].

The problem of misuse of language and concepts is crucial in neurorights. Many concepts usually used and associated with specific human rights, like ‘personal identity,’ ‘free will,’ ‘individuality,’ ‘agency,’ ‘self’ or ‘humanity’ itself, deal with the whole history of philosophical thought and seem to be too vague (on this topic, see [51,80,81]).

A second example of equivocal use of language is the recent term ‘technocratic oath,’ which may ‘help to establish core ethical principles that will ensure responsible innovation and to protect the fundamental human rights of patients and consumers in the use of neurotechnologies’ [82, p. 172]. The word ‘technocratic’ comes from the Greek τέχνη (*tékhnē*, ‘art, technique’) and κράτος (*krátos*, ‘power, dominion, government’): in this regard, *strictu sensu,* the word ‘technocratic’ means: ‘government by technicians.’ It is clear that the assonance with the Hippocratic oath was the inspiration for that oath. However, the very meaning of that term is the idea that the technicians governing humanity—in a kind of anti-democratic process—must take an oath.

Another linguistic concern emerges in some dangerous identifications implied in neurorights literature, like neural data with mental data [56] or the whole human being with his/her mind or brain [81]—as discussed above. On this point, we should remember that ‘the prime object of protection of the proposed rights are neither neurons, nor are the problems these rights address only caused by neurotechnologies, not even most of the time. Some of these rights—personal identity, freedom from algorithmic bias—do not seem to stand in any closer relation to neurons. The prefix “neuro” is thus a misnomer. … Most neurorights are variations of rights to the person’ [12]. Indeed, human rights are more than sufficient since they deal with the human being as a whole [83,84].

#### (iv) Another concern: from values to rights

As previously mentioned, it is possible that the attractiveness of neurorights among some legislators was fuelled by the political and social situation in Chile, namely, the ‘social outburst’ in Chile in 2019–2020, and the subsequent demand for a new Constitution. Thus, it is possible to interpret neurorights as one of many claims that emerged in a context where any moral value had to be transformed into a demand for public acknowledgement. On this topic, it is worth remembering that ‘interdisciplinary discussions on neurorights require clarity about rights. Moral values are often couched in the language of rights since rights have become a key currency in which societal conflicts are addressed and resolved’ [58, p. 465]. Indeed, as a society, we recognize the existence of many moral values that are desirable to the majority of citizens. This does not mean, however, that such values should also be recognized as rights or have their own legal status. It is necessary, therefore, to distinguish between what should be legislated about and what is good to educate the citizenry about so that they can make responsible use of the means at their disposal. In short, should we turn everything we consider morally good into law (or, better, a human right)? Indeed, ‘a central worry is the inflation of rights and their resulting devaluation. Human rights are powerful tools, they transform the legal landscape and potentially the lives of billions of people …. If every important interest or legitimate concern became a matter of human rights, they may lose their distinction, significance, and effectiveness, it is widely feared …. A parsimonious approach in loose analogy to Occam’s Razor should be adopted: human rights should not be multiplied without necessity’ [12].

### (b) Can legislation on neurorights end up harming science and medicine?

Chilean experience of legislation on medicine and the rights of patients has a sombre past (and present), which leads to fears that new protective legislation on the matters that concern us here may damage medical and scientific research, and end up harming those that it intends to protect [85]. We will briefly examine this previous legislation to explain what these fears are based on.

In 2012, law 29 584/2012, which ‘regulates the rights and duties of people in relation to actions linked to their health care’ entered into force in our country. According to Article 28 of this law, ‘no one with a psychological or intellectual disability who cannot express their wishes may participate in a scientific study’ [86]. The purpose of this clause was to protect the patients, who were unable to give their consent to participate in research projects, from possible abuse. Following a similar path to that of neurorights in the Chilean legislation, this provision of the law was discussed during the drafting process and different critics pointed out that, instead of protecting vulnerable people, it would end up excluding them from studies about these pathologies as well as from their potential benefits [87–90]. Indeed, in the worst possible scenario for biomedical research, the interpretation of this law by ethical committees and similar mechanisms spelled disaster for research into diseases in which ‘psychological disability’ could in some way be argued to exist. In fact, and with immediate effect, a dramatic drop was noted in the number of clinical trials registered in Chile on depression, bipolar disorder, schizophrenia, Alzheimer’s disease, Parkinson’s disease and the like. This reduction included clinical trials but also basic scientific and epidemiological studies [85,88].

In later years, far from improving, this dramatic situation deteriorated. In 2015, a new law in the Sanitary Code (Law 20 850 on the regulation of clinical studies) entered into force. Among its provisions is one that established the obligation of the grantee of the special authorization to conduct the study (whether a researcher or the university responsible, etc.) to answer for any harm caused ‘during’ the execution of the study, and not necessarily ‘as a result of it.’ Moreover, it enshrines a system of legal presumption and reversal of the burden of proof, with no time limit, together with a statute of limitations on legal responsibility dating from the appearance of the potential harm (possible ‘secondary effects’ of the drug), in effect making any time-bar on responsibility inapplicable. In an astonishing example of ‘tripping on the same stone,’ this over-protective measure caused a new dramatic reduction in clinical studies, now except for neuropsychiatric diseases. In short, the protective zeal of these articles ended up obstructing or simply ruling out research into diseases needing urgent attention.

Once again, in 2021, new amendments to Law 20 584 further restricted research by excluding from it those patients otherwise ‘healthy’ but ‘temporarily unable’ to express their wishes [86].

Even though today it is possible to quantify this fall in the number of registered clinical studies, the full harm done to research by the introduction of these laws is hard to calculate. We can only appreciate its scale first hand and estimate how many researchers and teams had to abandon their lines of research work. Likewise, the number of patients who may have ceased to benefit from participating in research projects and from the knowledge that they could have contributed is impossible to know, and the damage is probably irreversible.

We consider these legislative norms to exceed relevant international standards and to be contrary to the Helsinki Declaration, which states that ‘medical research with a vulnerable group is only justified if the research is responsive to the health needs or priorities of this group and the research cannot be carried out in a non-vulnerable group. In addition, this group should stand to benefit from the knowledge, practices or interventions that result from the research’ [91]. Along the same lines, the Council for International Organizations of Medical Sciences prescribes for vulnerable persons and groups that: ‘Research ethics committees need to be sensitive to not overtly excluding [vulnerable] people, and allow them to participate by requiring that special protections be put in place’ [92]. The logic promoted by these guidelines is that vulnerable groups must be included in research related to their health unless a good scientific reason justifies their exclusion [92].

This was the context in which neurorights made their appearance on the scientific stage in Chile. The law of neurorights has a series of characteristics whose altruistic purpose is obvious. However, part of its provisions (some of which refer directly to the aforementioned Law 20 584) are very similar to the earlier laws that have already severely hindered research in Chile. By attempting to regulate, but finally explicitly restricting neurotechnologies, and in the light of previous local experience, it is reasonable to fear that the neurorights law will have the same deleterious effect, this time on BCIs, NF and other neurotechnologies, in a broad definition of the term. Excessive regulation could once again prompt ethics committees and regulatory bodies to be over-restrictive and finally discourage scientists in this field.

To give one example, the problem of ‘consent’ can be extremely complex [53]. In effect, many of these technologies *must* be applied to patients who are unconscious and unable to consent. For example, neuromonitoring during anaesthesia in intraoperative processes is a neurotechnology that simultaneously *decodes* and *modulates* the cerebral cortex to ensure that the patient’s sensory and motor functions remain unimpaired in the operating theatre; it can only be applied when the patient is unconscious and informed consent impossible to obtain. Recently, local ethics committees blocked the acquisition of clinical data and blood samples from patients suffering from lung disease caused by Hanta virus owing to the ‘temporary’ inability of the patients to give consent (based on the laws previously mentioned), despite the fact that there is no other way of accessing these data or of ‘anticipating’ the inability to consent, this being an illness of sudden and unpredictable onset (and that these laboratory data could have been tremendously necessary for future sufferers from a serious disease that is endemic in Chile). Fortunately, after an appeal, this restriction was overturned (M. Ferrés 2023, personal communication).

Furthermore, the paradigmatic condition for the clinical application of BCIs, as mentioned previously, is the locked-in state of patients with ALS, who are unable to communicate with their surroundings and are naturally unable to offer their informed consent. In the worst possible scenario, and owing to the regulations already approved and the new neurorights, will Chile become the first country whose legislation rules out the possibility of communicating via a BCI with a patient affected by this pathology?

We believe that a law which ‘limits’ the use of a certain technology should be precise and clear. It should avoid being ‘over-inclusive,’ thereby inhibiting as an ‘undesired secondary effect’ the use and development of potential technologies necessary for clinical medical use and scientific research. Some of its provisions lack linguistic rigour owing to the use of open and undefined terms that regulate key matters, including the purposes of the law. For further information on the potential problems of the neurorights law approved in Chile, please see Annex 2 in the electronic supplementary material.

We believe that if we are not careful, and without suitable amendments to previous laws, another over-protective law could affect the development of neurotechnologies urgently needed in our country (as they are everywhere). In the fear of potential malicious intent, neuroright laws might discourage and curb the very research that might indeed advance and develop BCI/NF science and technology. In a sombre scenario, it could frustrate the development in Chile of all future technologies still not created, limit the participation of Chilean research teams and patients in multicentric studies, and end up being a ‘nail in the coffin’ of scientific development in our country.

In August 2023, Chile became the first country in the world whose Supreme Court granted a plaintiff’s writ to protect their constitutional rights against a private Unite States-based company that markets a home BCI device for accessing and storing ‘private neuronal data’ (electrical brain data) [93]. Of course, nothing justifies an eventual violation of the privacy of patients or users of any technology. However, is this good news? Or is it a fateful omen for the future development of medical and scientific neurotechnologies in our country?

## 3. Concluding remarks

Neurorights are becoming an important topic in the scientific community and increasingly in the wider public. It is possible that in the coming years, they will be discussed and eventually included in legislations other than the Chilean (e.g. in Spain [13]). In this regard, the present article disentangles some of the epistemological and practical concerns regarding neurorights, pointing out a number of possible detrimental consequences of such a legislation—mainly referring to the Chilean experience. These consequences may end up blocking neuroscientific research. Finally, the paper argues that rights should not be multiplied without necessity. A careful reconsideration of human rights may be sufficient to face the concerns emerging from neurotechnologies (e.g. BCIs) and/or creating (or enhancing existing) guidelines in the clinical field: this would not harm clinical investigation and might be enough to provide a clearer and more defined legal and ethical background.

The Chilean case makes us reflect on the need for better communication between politicians (or legislators) and scientists (in this case, neuroscientists): as mentioned above, this is a pressing necessity, since scientific advances are systematically impacting essential aspects of human life and coexistence.

Just as the maxim ‘*primum non nocere*’ (first, do not harm) is applied to medicine, we believe that the same logic should govern laws on health and that extreme care should be taken to protect personal rights without delaying scientific progress.

## Data Availability

Electronic supplementary material can be found at [94].
